# Professor Rolfe Birch: A Pioneer in Peripheral Nerve Surgery and a Mentor to Generations

**DOI:** 10.7759/cureus.74030

**Published:** 2024-11-19

**Authors:** James Bennett, Edward Karam, Kapil Sugand, Anthony Kinnair, Dennis Hazell, Ashley Simpson, Anna Panagiotidou, Michael Fox, Marco Sinisi

**Affiliations:** 1 Peripheral Nerve Injury, Royal National Orthopaedic Hospital, London, GBR

**Keywords:** brachial plexus injury, historical vignette, obituary, peripheral nerve injury, rolfe birch

## Abstract

Professor Rolfe Birch, a founding head in the field of peripheral neurological surgery, left an enduring legacy marked by groundbreaking research, innovative surgical techniques, and unwavering dedication to patient care. From his early fascination with the nervous system to his pioneering work in brachial plexus injuries and the establishment of the UK's (if not the continent’s) first peripheral nerve injury unit, Prof. Birch's contributions transformed the landscape of understanding, diagnosing, and treating peripheral nerve pathologies. His enthusiasm and teaching style had a profound impact on inspiring generations of future surgeons from interface specialties including orthopedics, plastics, vascular, and neurosurgery. This article aims to reflect on his life and his work to advance the understanding and treatment of complex nerve injuries.

## Introduction and background

Early life, education, and the spark of inspiration

Born in Scotland in 1944, Professor Birch was the son of a toolmaker and an elementary school teacher. His education at Haberdashers' Aske's Hampstead and medical studies at Cambridge University further nurtured his academic pursuits, culminating in his ongoing medical training at St George's Hospital in the late.

It was during the early 1970s on the Southwest London Orthopaedic training scheme that Prof. Birch's path toward peripheral nerve surgery began. For his fervor for this niche field, he was awarded a six-month scholarship in microsurgical training and laboratory research. This pivotal experience ignited his fascination with the intricacies of the nervous system and the potential of microsurgery to revolutionize the treatment of nerve injuries.

A career dedicated to innovation and patient care

The skills gained from his scholarship earned Prof. Birch a post as a consultant orthopedic surgeon at St Mary’s Hospital, Paddington, England, and the Royal National Orthopaedic Hospital, Stanmore, England, in 1979. Here, he collaborated with the esteemed Mr. George Bonney, who worked together to develop their research and propose their classification in nerve injuries as either conduction block or degenerative lesions. This simplified the classification further from that previously proposed by Seddon and Sunderland [1,2]. Upon Mr. Bonney’s retirement in 1991, Prof. Birch moved his clinical unit to the Royal National Orthopaedic Hospital, where his academic and clinical work flourished, as he went on to produce many of his published works during his time here. He also further strengthened the working relationship with colleagues at Queen’s Square, the National Hospital for Neurology and Neurosurgery, and Hammersmith Hospital. His collaborative spirit fostered a conducive environment for effective exchanges, transparency, and open channels of communication leading to advancements in the field.

In 2003, he was appointed consultant-in-charge at the War Nerve Injuries Clinic, Defence Medical Services Rehabilitation Unit, Headly Court. He was also appointed as a professor of peripheral neurological surgery at the University College of London. In spite of retiring from the National Health Service (NHS) in the late 2000's, Prof. Birch continued to run this clinic and contributed to the research of the department that he founded for several years afterward. Prof. Birch passed away peacefully on May 22, 2023, survived by his partner and children. His funeral was attended by colleagues, family, and friends from all over the world just to pay their last respects.

## Review

Academic work

His numerous published works, including his textbook, "Surgical Disorders of the Peripheral Nerves" (2011), remain a cornerstone of the field, providing guidance to practitioners worldwide regarding the diagnosis, treatment, and rehabilitation of these complex injuries [3]. The book is written in a unique conversational style and is affectionately referred to by surgeons in the field as “The Bible” of peripheral nerve surgery. Through meticulous research and clinical practice, Prof. Birch demonstrated the efficacy of early surgical intervention, leading to improved functional outcomes and quality of life for countless patients. His work challenged the traditional approach of delayed surgery and advocated for timely intervention in view of attaining superior clinical outcomes [4,5].

Prof. Birch’s body of work can be largely categorized into the following topics:

Classification and Assessment

Prof. Birch also contributed to the development of classification systems for peripheral nerve injuries, which enabled greater clarity and standardization in the field. These systems help facilitate diagnosis, treatment planning, and communication among healthcare professionals, ultimately benefiting patients. He also recognized the need for standardized assessment tools to evaluate nerve function and monitor recovery, enabling clinicians to track progress and make informed decisions [2,3]. Prof. Birch's work in this area has helped to streamline and optimize the management of peripheral nerve injuries, improving communication among healthcare professionals and ultimately benefiting patients.

Brachial Plexus Injuries

Prof. Birch's work on brachial plexus injuries transformed the landscape of treatment for these injuries, often caused by traction in high-energy trauma such as motorcycle accidents or falls from heights [6,7]. He meticulously refined surgical techniques, including nerve repair, grafting, and transfers, as well as tendon transfers, to restore function and improve quality of life [8]. Prof. Birch's contributions instilled hope in patients who previously faced limited options and significantly improved their chances of regaining functionality and independence.

Nerve Regeneration

Prof. Birch explored the intricate mechanisms of nerve regeneration, conducting detailed research into the role of growth factors, neurotrophic agents, and other factors in promoting nerve repair. This laid the groundwork for future therapeutic interventions with far-reaching implications for standard clinical practice and areas of new research in the broader field of peripheral nerve injuries. This included Prof. Birch's work contributing to the development of new treatments, such as gene therapy and stem cell therapy [9].

Microsurgery and Nerve Transfers

Prof. Birch was a master of microsurgery, a technique requiring exceptional skill and precision to operate on delicate nerve structures. He recognized its potential to revolutionize peripheral nerve repair, and his expertise in nerve grafting and nerve transfers enabled him to restore function even in cases of severe nerve damage, reconnecting severed nerves or rerouting healthy nerves. Prof. Birch's surgical ability and meticulous attention to detail inspired countless surgeons and continue to shape the practice of peripheral nerve surgery today, pushing the boundaries of what is possible in nerve repair [10,11].

Obstetric Brachial Plexus Injury (OBPI)

**Figure 1:**
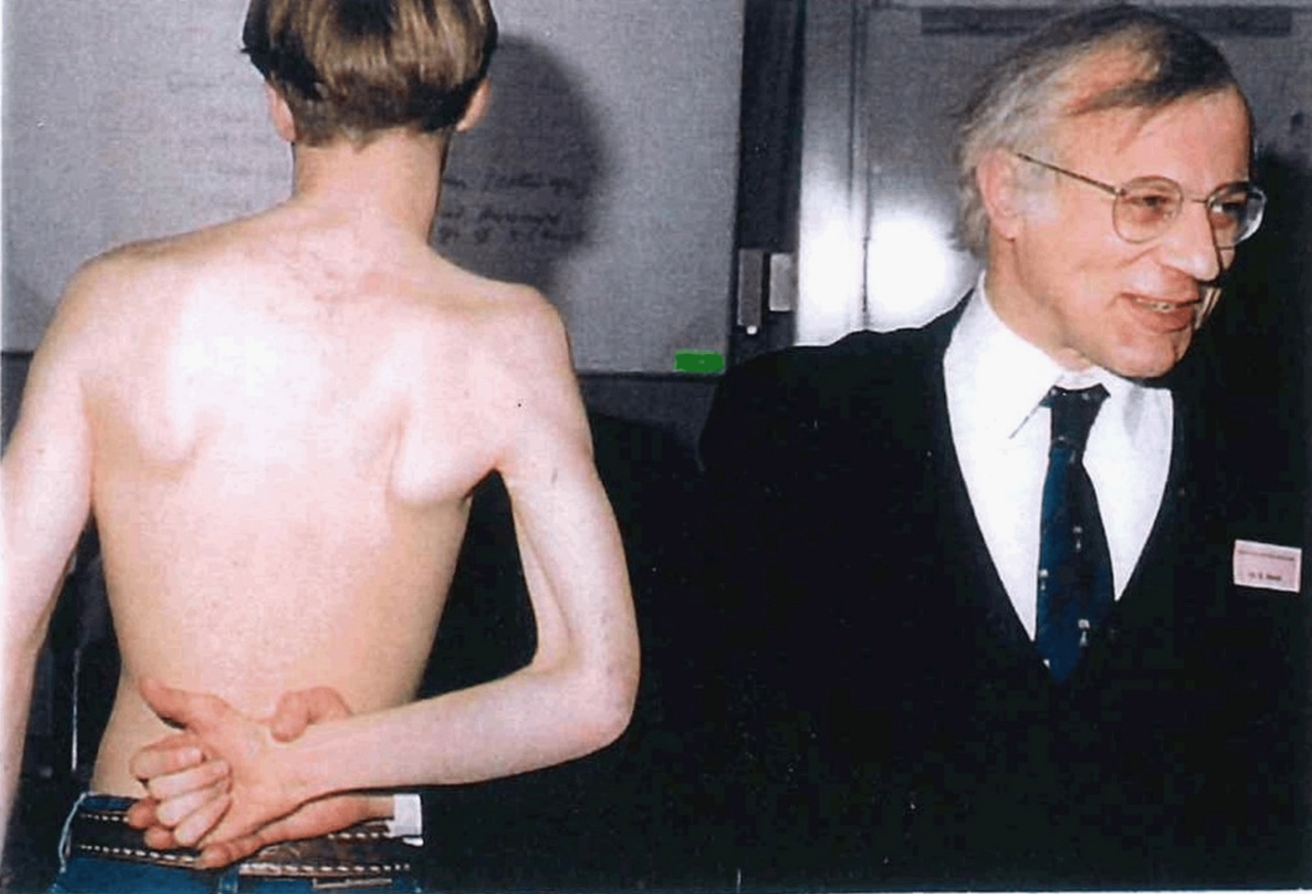
Professor Birch demonstrating examinations of obstetric brachial plexus injuries at one of several workshops in Heerlen, the Netherlands, between 1991 and 1998. Source: Photograph on display in, and property of, the peripheral nerve injury unit building, Royal National Orthopaedic Hospital, Stanmore, England. Permission to reproduce this image has been obtained.

With an incidence of 0.5-3 per 1000 births, this is a hugely distressing subject for any parent and Prof. Birch understood the importance of reassuring parents and working with children to overcome their injuries [12]. In particular, some of his work focused on highlighting the differences between the types of neurological lesions suffered by infants and adults [13]. OBPI was a large part of his teachings, particularly at international conferences and workshops (see Figure 1 below).

Prof. Birch also shed light on the issue of iatrogenic nerve injuries. His research, including publications like "Iatrogenic injuries of peripheral nerves" (1991) and "Latropathic injuries of peripheral nerves" (2001), highlighted the importance of preventative measures and early treatment to minimize the impact of these injuries [14,15]. Prof. Birch was particularly aware of the emotional distress surgeons can suffer when feeling responsible for an iatrogenic injury. His letters would often state how the intricacies of the operation can make it challenging to avoid causing a peripheral nerve injury, with his supportive tone focused on supporting the surgeon whilst keeping the patient’s best interests at heart. Prof. Birch's advocacy for patient safety and his efforts to raise awareness of this important issue have undoubtedly prevented countless nerve injuries and improved the quality of care for patients worldwide.

Mentorship and legacy: inspiring generations

Prof. Birch's mentorship extended beyond the clinical and academic; he instilled in his trainees a deep sense of compassion, dedication, and commitment to excellence in patient care. Before the dawn of social media and channels of publicity, his reputation preceded him to the extent that surgeons from every corner of the world would attend fellowships just to learn from the master himself.

He was known for his precise, sharp dissection of peripheral nerves and unparalleled understanding of their anatomy. His teaching rounds at Stanmore were legendary, filled with insightful questions and gentle encouragement that pushed trainees to reach their full potential.

Prof. Birch's sense of humor and humility endeared him to colleagues and patients alike, with his interactions marked by attentiveness and compassion. He spoke to everyone he encountered in life as if they were the most important person to him at that particular moment, a trait that left a lasting impression with patients enquiring about him long after his retirement.

## Conclusions

Professor Rolfe Birch's legacy as a pioneer in peripheral nerve surgery is undeniable. His charm and spirit, coupled with his unwavering dedication to patient care and research, transformed the landscape of this modern and continually evolving surgical specialty. As the founding father of the Peripheral Nerve Injury Unit at the Royal National Orthopaedic Hospital, the unit remains an international beacon for managing complex nerve pathologies approached by interface surgical specialties from orthopedics, plastics, vascular, and neurosurgery. His contribution to literature is still considered the gold standard for future generations of surgeons to continue delivering the highest quality of patient care long beyond his clinical practice.
